# Contrasting Diversity Patterns of Crenarchaeal, Bacterial and Fungal Soil Communities in an Alpine Landscape

**DOI:** 10.1371/journal.pone.0019950

**Published:** 2011-05-12

**Authors:** Lucie Zinger, David P. H. Lejon, Florence Baptist, Abderrahim Bouasria, Serge Aubert, Roberto A. Geremia, Philippe Choler

**Affiliations:** 1 Laboratoire d'Ecologie Alpine, CNRS-UMR 5553, Université de Grenoble, Grenoble, France; 2 Station Alpine J. Fourier, CNRS-UMS 3370, Université de Grenoble, Grenoble, France; Argonne National Laboratory, United States of America

## Abstract

**Background:**

The advent of molecular techniques in microbial ecology has aroused interest in gaining an understanding about the spatial distribution of regional pools of soil microbes and the main drivers responsible of these spatial patterns. Here, we assessed the distribution of crenarcheal, bacterial and fungal communities in an alpine landscape displaying high turnover in plant species over short distances. Our aim is to determine the relative contribution of plant species composition, environmental conditions, and geographic isolation on microbial community distribution.

**Methodology/Principal Findings:**

Eleven types of habitats that best represent the landscape heterogeneity were investigated. Crenarchaeal, bacterial and fungal communities were described by means of Single Strand Conformation Polymorphism. Relationships between microbial beta diversity patterns were examined by using Bray-Curtis dissimilarities and Principal Coordinate Analyses. Distance-based redundancy analyses and variation partitioning were used to estimate the relative contributions of different drivers on microbial beta diversity. Microbial communities tended to be habitat-specific and did not display significant spatial autocorrelation. Microbial beta diversity correlated with soil pH. Fungal beta-diversity was mainly related to soil organic matter. Though the effect of plant species composition was significant for all microbial groups, it was much stronger for *Fungi*. In contrast, geographic distances did not have any effect on microbial beta diversity.

**Conclusions/Significance:**

Microbial communities exhibit non-random spatial patterns of diversity in alpine landscapes. Crenarcheal, bacterial and fungal community turnover is high and associated with plant species composition through different set of soil variables, but is not caused by geographical isolation.

## Introduction

Microorganisms play a key role in biogeochemical cycling and ecosystem functioning [1], [2]. Understanding and predicting the spatial distribution patterns of microbial communities is crucial to anticipate ecosystem responses to global changes [2]. Although these questions are extensively addressed for macro-organisms [3], microbial biogeography gained renewed interest only recently with the advent of molecular tools. Based on these molecular techniques, some studies provided evidence for habitat determinism (e.g. salinity, pH) on microbial community distribution regardless of geographic location [4], [5]. This support the Baas-Becking hypothesis “everything is everywhere, but, the environment selects” [6], which assumes large dispersal potential and low extinction rate for microbes. This hypothesis has been questioned with several observations of increasing microbial community divergences with increasing geographic distances, hence suggesting a microbial provincialism (reviewed in [7], [8]). The inconsistency of the results on that topic still fuels the debate, but might actually arise from differences in the spatial and taxonomical scales considered, as suggested for macroorganisms and individual bacterial species [9], [10].

Soils are heterogeneous systems composed of highly diverse microhabitats that may form complex spatial patterns in soil microbial communities. At the landscape scale, these patterns have been suggested to be driven by plant communities [11], [12], [13], [14]. Indeed, plant species exhibit a variety of root architectures, metabolism and growth strategies that affect the quality and quantity of soil organic matter (SOM) through litter deposition and root exudation [15], [16]. Furthermore, the rhizosphere carbon flow provides high amounts of diverse organic substrates, and includes signal molecules that may regulate the population density of soil microbes [17]. Because of the importance of mutualistic/parasitic interactions described between plants and microbes and among microbial foodwebs, one may expect a strong effect of individual plant species on soil fungal [18], and bacterial community composition [19]. This effect is, however, not always observed [14], [20], [21]. Numerous studies have reported soil pH, and nutrients availability and quality as main drivers of soil microbial community composition [5], [22], [23], [24]. Both of these factors are known to be influenced by vegetation [16]. Spatial covariation between plant community and microbial communities has been reported too [20], [25], [26], [27], [28]. Most of these studies have failed to identify the relative contributions of soil properties, plant cover, and isolation by distance in the spatial patterning of soil microbes (but see [28] for *Bacteria*).

Usually, investigations carried out on soil microbial biogeography focus on only one microbial domain (but see [21], [27], [29]). However, *Bacteria*, *Archaea* and *Fungi* are essential actors interacting in the soil food web, and their response to plant cover might differ. Indeed, some studies suggest that *Fungi* are more tightly associated with plants than prokaryotes, the latter being more influenced by soil properties [14], [27]. Furthermore, although many *Fungi* and *Bacteria* compete for the same resources [30], *Fungi* can degrade complex molecules from plant litter that are inaccessible for most bacteria [31], [32]. These apparent contrasting ecological requirements may affect beta diversity patterns of these two microbial domains [27]. However, a comprehensive study examining this at the landscape scale has not been done so far.

**Figure 1:**
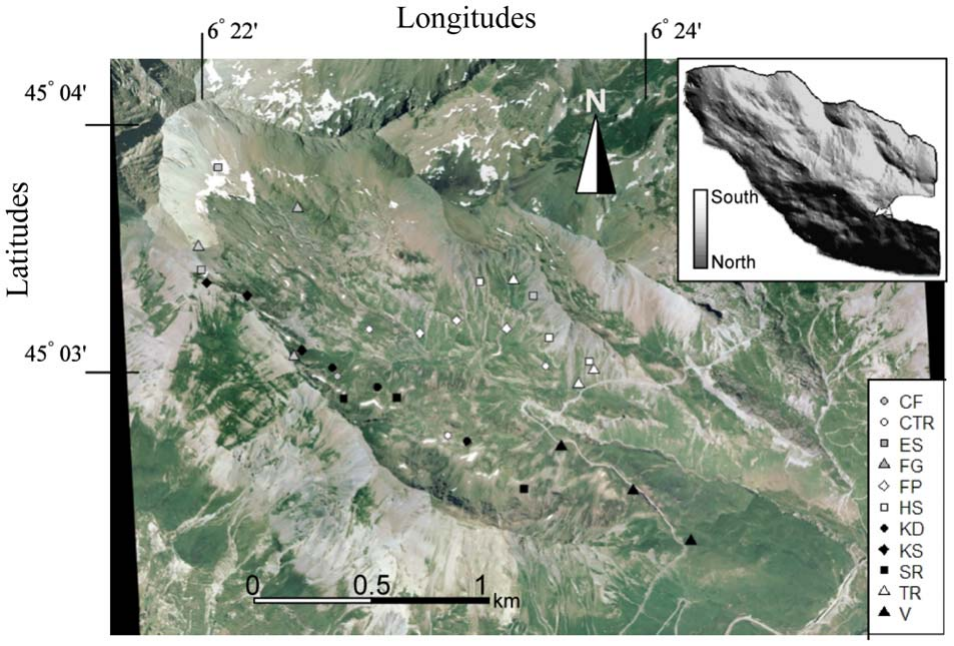
Aerial photograph of the investigated area and location of sampling units (Vallon de Roche-Noire, massif du Galibier, France). The top-left inset shows slope aspect with a gray-colour palette indicating North/South exposure. Symbols correspond to the eleven habitat types. See Table 1 and Table S1 for the dominant plant species and the environmental characteristics of each habitat.

High-elevation environments provide a unique opportunity to assess the underlying factors of spatial patterning of microbial communities, as steep environmental gradients determine high turnover in plant species composition over short distances [33], [34], [35]. Previous studies highlighted the striking dissimilarities in microbial community composition of neighbouring early and late snow-melting sites [36], [37] or along vegetation gradients [25], [38] in alpine tundra. In this study, we investigated soil microbial communities at thirty-three sites representing eleven contrasting habitat types of an alpine landscape (Fig. 1). Microbial communities were characterized by means of Capillary Electrophoresis Single Strand Conformation Polymorphism (CE-SSCP) based on rRNA genes. Based on this data set, we addressed the following questions: (i) How do archaeal, bacterial and fungal soil communities change across this alpine landscape? (ii) What are the relative contribution of plant community composition, environmental conditions, and geographic isolation on microbial beta diversity patterns? (iii) Do the three microbial domains respond similarly to these environmental drivers?

**Table 1:** Dominant plant species in the eleven investigated habitats.

Name	Dominant species
CF	*Carex foetida, Alchemilla pentaphyllea, Salix herbacea*
CTR	*Carex sempervirens, Trifolium alpinum*
ES	*Crepis pygmeae, Doronicum grandiflorum*
FG	*Festuca violacea, Alchemilla filicaulis, Geum montanum*
FP	*Festuca paniculata*
HS	*Helictotrichon sedenense, Festuca violacea*
KD	*Kobresia myosuroides, Dryas octopetala*
KS	*Kobresia myosuroides, Sesleria coerulea, Carex rosae*
SR	*Salix retusa, Salix reticulata*
TR	*Trifolium pratense, Geranium sylvaticum*
V	*Vaccinium uliginosum, Vaccinium_myrtillus*

See Fig. 1 for the sampling unit (SU) locations.

## Results

### Vegetation and environmental characteristics of the studied area

**Figure 2:**
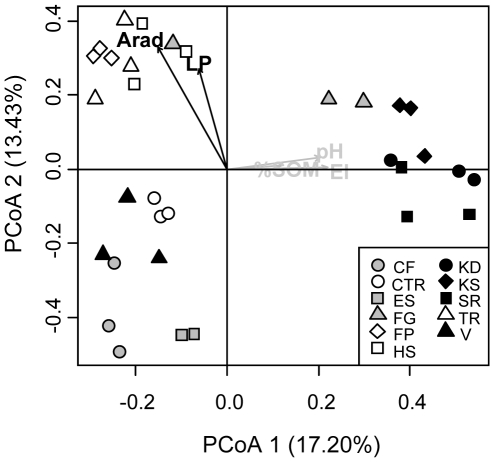
Principal Coordinate Analysis (PCoA) of the vegetation-dissimilarity matrix and vector fitting of the environmental variables. The variation explained by the axes is indicated. Gray arrows and black arrows represent non-significant and significant fittings, respectively (Bonferroni-corrected *P*<0.05). El: elevation; Arad: annual radiation; pH: soil pH; %SOM: % soil organic matter; LP: live phytomass.

The dominant plant species and the environmental characteristics of each habitat type are given in Table 1 and Table S1, respectively. The PCoA ordination of the vegetation-dissimilarity matrix showed a marked contrast between north-facing (SR, KS, KD and to a lesser extent FG) and south-facing slope habitats (FP, HS, TR; Fig. 2a). A third group (V, CF and CTR) corresponded to habitats with the most acidic soils (Fig. 2, Table S1). The projection of environmental variables onto the vegetation-PCoA ordination confirmed these results (Fig. 2a). Annual radiations (Arad), live phytomass covaried with plant community composition. Given the low variance explained by the first two PCoA axes, we also carried out Mantel tests between vegetation-dissimilarity matrix and canonical distance matrices obtained for each environmental variable. Using this approach, plant species composition significantly covaried with Arad, soil pH, and SOM (Spearman rank ρ = 0.21, 0.41 and 0.37 respectively, Bonferroni-corrected *P*<0.05).

### Microbial community responses to the biotic and abiotic context

**Figure 3:**
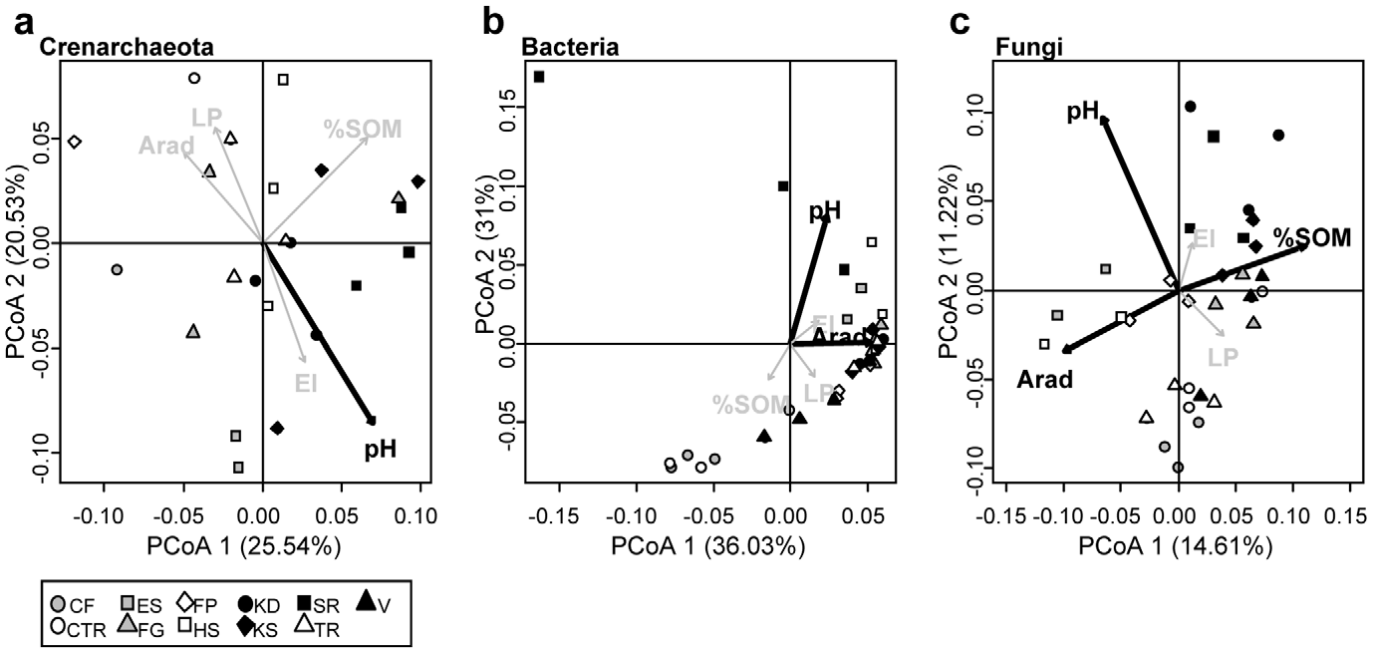
Principal Coordinate Analysis (PCoA) of crenarchaeal (a), bacterial (b) and fungal (c) dissimilarity matrices and vector fitting of the environmental variables. Gray arrows and black arrows represent non-significant and significant fittings respectively (Bonferroni-corrected *P*<0.05). See Fig. 2 for abbreviations.

PCoA ordinations of microbial-dissimilarity matrices correlated with soil pH for all microbial domains (Fig. 3). Crenarchaeal communities from V, CTR, FP and CF soils are not represented on the PCoA ordination because of PCR-amplification difficulties. Bacterial beta-diversity was also related to annual radiation (Fig. 3b) and fungal beta-diversity was further associated with SOM (Fig. 3c). Given the first two PCoA axes obtained for *Fungi* explained a little amount of the total variance, Mantel tests were also performed between fungal-dissimilarity matrix and canonical distance matrices of each environmental variable. This analysis confirmed the vector fitting results (Spearman rank ρ = 0.14, 0.31 and 0.15 for Arad, soil pH and SOM respectively, Bonferroni-corrected *P*<0.05).

**Figure 4:**
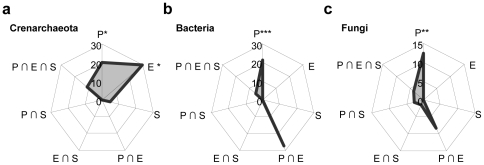
Pure and combined effects of plant species composition (P), environmental variables (E) and geographic distances (S) on crenarchaeal (a), bacterial (b), and fungal (c) communities. Values indicate the percentage of variance explained by each effect. The total percentage of variance explained by the model was: 42.0%, 51.4%, and 26.4% for *Crenarchaeota*, *Bacteria*, and *Fungi*, respectively. Significance of pure effect was tested using partial db-RDAs with 1000 Monte Carlo permutations and is indicated as in Table S2. Significance tests for combined effects are not available [59].

We partitioned the variation in microbial community dissimilarity matrices into components that accounted for vegetation, environment and geographic distance (i.e. pure effects) and their combined effects (Fig. 4, Table S2). All the models were significant and explained more than 26% of the microbial community variation. These models were poorly sensitive to the number of selected PCoA axes for vegetation (Fig. S1b–d). The pure effect of plant communities was significant for the three microbial domains whereas the pure effect of the environment was significant only for *Crenarchaeota.* Patterns of bacterial beta diversity were better accounted for the combined effect of plant communities and environment. In the case of *Fungi*, the pure effect of plant communities was the most significant descriptor. The pure effect of geographic distances did not significantly explained microbial community variation, neither globally (Fig. 4), nor at different spatial scales (Fig. S2). Geographic distances contributed to microbial community variation only when considered in combination with the other descriptors.

## Discussion

Although significant efforts have been made to determine drivers of microbial biogeography [4], [5], [7], [8], [13], there is still a lack of studies assessing the relative contribution of different set of descriptors (biotic and abiotic) on the landscape scale diversity patterns of the *Crenarchaeotes, Bacteria* and *Fungi*. Our study aimed to bridge this gap by focusing on an alpine landscape.

The investigated habitat types corresponded to markedly different plant communities even if distances between sampling sites were small (Fig. 1–2, Table S1). The turn over in plant species composition is high and partly explained by elevation and topography [34], [35]. Community scale descriptors, *i.e.* soil pH and SOM, significantly covaried with plant communities. Superficial soils with sparse vegetation cover (*i.e.* ES, HS, SR) exhibited higher pH, and lower level of SOM than deeper and more mature soils covered by denser vegetation (*i.e.* CF, CTR, V, FP; Fig. 2a, Table S1). The same changes in soil-vegetation properties have been described along receding glaciers chronosequences [39]. Plant species turnover affects soil nutrient availability and soil properties by varying quality and quantity of litter fluxes and root exudates [15], [16]. Therefore, there is a complex intricacy between climate, topography, plant species composition and soil properties, and the partitioning of microbial diversity with respect to these different descriptors has to consider both pure and combined effects.

The effect of vegetation and environmental variables on microbial community composition differed among the three microbial domains investigated (Fig. 3). Fungal community dissimilarity patterns were related to SOM (Fig. 3c), which is in line with the typical saprophytic status of most of *Fungi* and their higher competitiveness for complex substrates compared to *Bacteria* [31], [32] The observed SOM effect on fungal communities may be direct, but may also reflect the recruitment of different mycorrhizal association type in ecosystems displaying contrasted SOM and nutrient cycles. Indeed, ericoid- and ectomycorrhizal associations are usually more occurrent in ecosystems with lower SOM recycling [40]. Crenarchaeal communities were not affected by SOM (Fig. 3a), which supports the idea that many soil *Crenarchaeas* are autotrophic ammonia oxidizers [41]. This may also explain the lack of creanarchaeal community covariation with annual radiation compared to bacterial and fungal communities (Fig. 3b–c). Indeed, annual radiation is a decisive factor for plant growth and growing season length that strongly impacts on plant community composition (Fig. 2), nutrient conservation strategy [33], and therefore on soil resources [16], [40]. In contrast, annual radiation did not covaried with soil water content at the sampling time (data not shown), excluding an immediate effect of water availability on the observed patterns. Both cases suggest a direct or indirect effect mediated by plant communities on bacterial and fungal communities. In general, soil pH appeared to be a good predictor for microbial community composition as reported previously [5], [22], [23], [42]. For *Crenarchaeota*, Nicol *et al.* (2008) observed that different creanarchaeal lineages occurred in soils with pH that varied by 2.5 units. In our study, crenarchaeal 16S RNA genes were hardly PCR-amplifiable in the most acidic soils (CF, CTR, and FP, Table S1). In contrast, bacterial and fungal PCR amplifications were possible for these samples, which suggests a possible detrimental effect of low pH on crenarchaeal populations. The significant covariation between pH and bacterial diversity may be related to *Acidobacteria*, a dominant group of soil *Bacteria* [43] known to be highly responsive to soil pH [22]. On the other hand, variation in soil pH may reflect differences in the availability of simple organic substrates [16] for which *Bacteria* are more competitive [12], [31]. It is generally considered that fungal communities are less sensitive to soil pH than bacterial communities due to their wider pH range for optimal growth [17], [23]. However, arbuscular mychorrizal fungi biomass has been reported to co-vary with soil pH [25]. This might explain why we also found significant covariation between *Fungi* and soil pH.

In this study, we used variation partitioning [44] to disentangle the relative contribution of the different drivers of microbial diversity. We found that the pure effect of plant species composition was always significant regardless of microbial taxa (Fig. 4). Plants may affect microbial assemblages either through specific mutualistic/pathogenic interactions, soil structure changes *via* varying root architectures, specific root exudates, or through differences in competition intensity for nutrients [11], [13], [18]. The combined effect of environmental variables and plant community composition noticeably explained bacterial and fungal community variation (Fig. 4b–c), implying that soil pH and/or SOM indirectly affect these communities (Fig. 3b–c). This latter result shows that the plant-soil feedbacks strongly act on microbial community assemblages, mainly through variations in mutualistic associations with plants for *Fungi*, and plant-mediated modification of soil properties for *Bacteria*. Crenarchaeal communities were rather explained by environmental conditions, mainly due to the soil pH effect (Fig. 3a) as reported previously for autotrophic ammonia oxidizer *Crenarachaeota* [42]. Finally, none of the microbial beta diversity patterns were due to geographic distances, neither at the landscape scale (Fig. 4, Fig. S1), nor for different classes of spatial distances (Fig. S2). This result provides evidence that geographic distances do not account for microbial community changes across the landscape. This opposes previous findings for *Bacteria* and *Fungi* at large spatial scales [7], [8], or for individual bacterial taxa at local scales [10], [38]. Possibly, these contradictory observations result from differences in taxonomic resolution. Mostly based on sequencing approaches, these other studies reported patterns at the “species” level, whereas our method of investigation was based on fingerprinting, which provides a fuzzy, yet consistent, picture of local microbial communities. Moreover, the matter of spatial scale is acknowledge to be of primary importance in pattern detection [9], and the distances considered in our study (up to 1000m) are smaller than the one usually considered [7], [8]. Taken together, this suggests that either the spatial scale of the study area was too small to observe isolation by distance or our taxonomic resolution was too coarse to detect such an effect. Further studies based on sequencing approaches are needed for clarification.

A main finding of this study is that, although the landscape scale beta diversity patterns of the three microbial domains investigated are all related to plant community composition, they result from different set of biotic and abiotic factors (Fig. 4). Crenarchaeal community variation is mainly explained by the pure effect of environmental factors (Fig. 4a). Bacterial community assemblages covary with plant species composition and with the combined effect of plant and environment (Fig. 4b). Finally, the most striking feature of fungal community diversity pattern is its strong correlation with changes in plant species composition (Fig. 4c). This is in line with other studies reporting a higher responsiveness of fungal diversity to plant species identity compared to prokaryotic diversity [14], [27].

To our knowledge, our study is the first to provide a comprehensive view of the landscape scale patterns of alpine soil microbial communities. We estimated the relative contribution of different drivers on these patterns using variation partitioning of microbial beta diversity matrices. Although purely correlative, our findings help us to sharpen our hypotheses on the distribution of microbes, and provide a set of potential indicators for predicting microbial community composition in alpine soils. Further studies such as experimentally manipulating of plant communities and/or the environment and studies with a higher taxonomic resolution will provide even better insights into the underlying mechanisms involved in the spatial distribution of soil microbial communities.

## Materials and Methods

### Ethics Statement

Sampling was conducted in a non-protected area, with the approval of the commune of Le Monêtier-les-Bains, owner of the field.

### Study area and soil sampling

The study area is located in the Grand Galibier Massif in the French South-Western Alps (Vallon de Roche Noire, commune de Le-Monêtier-les-bains, France; 45°0.05′N, 06°0.38′E). The area is a high-elevation watershed, ranging from 1,900 to 2,800 m, the main slopes facing Southwest and Northeast (Fig. 1, Table S1). Vegetation is composed of a mosaic of herbaceous and heath communities. The area is slightly grazed by sheep at the end of the summer. Based on previous vegetation studies [45], we selected eleven types of habitats that encompass most of the landscape heterogeneity. Habitat types are characterized by a combination of topographic variables and vegetation (Fig. 1 and Table 1). Each habitat was named after the dominant vascular species, usually a grass, sedge or shrub species (Table 1). Three Sampling Units (only two for ES), hereafter SU, were selected per habitat. SUs of a given habitat were separated by at least 100 m. The shortest distance between any two SUs was calculated and a geographic distance matrix was created. In each SU, three 10 cm deep soil cores were sampled in a homogeneous 5×5 m plot. Soil core collection was carried out at the peak of standing phytomass in mid July 2007. All soil samples were sieved to 2 mm, and kept at −20°C for subsequent analysis.

The floristic composition of each SU was assessed by visual estimate of the percentage cover of vascular plant species in the 5×5 m plot. We constructed a floristic table including the cover of a total of 191 species from which we disregarded the rarest ones, i.e. species with fewer than three occurrences in the whole data set. We estimated floristic dissimilarities between SUs using the Bray-Curtis index. The resulting distance matrix is hereafter called the vegetation-dissimilarity matrix.

For each SU, the following topographical variables were estimated: slope, exposure and elevation. Interpolated climatic variables for the study area (temperature, precipitation, annual radiation) were retrieved from the meteorological model Aurelhy (Météo-France, [46] downscaled at a 100 m resolution. For further analyses, we retained the two uncorrelated variables, namely Elevation (El) and Annual Radiation (Arad). In each SU, the peak standing crop phytomass was collected in three plots of 20×20 cm except in FP, HS, and V where 50×50 cm plots were used. Live material (LP) were dried at 85°C for 48 hours and weighed. LP values were log-transformed for subsequent analyses. Soil pH was measured after mixing 5 g of soil with 12.5 ml of distilled water [47]. The Soil Organic Matter content (SOM) was determined by loss-on-ignition [48]. El, Arad, SOM, pH and LP were used to estimate the environmental distances between SUs. Data were normalized data and the canonical distances were calculated. The resulting distance matrix is hereafter called the environmental-dissimilarity matrix.

### Microbial community analyses

Soil DNA extractions were carried out in triplicates from 0.25 g wet mass of each soil sample with the PowerSoil-htp^TM ^96 Well Soil DNA Isolation Kit (MO BIO Laboratories, Ozyme, St Quentin en Yvelines, France) according to the manufacturer's instructions. DNA concentration was quantified using the NanoDrop ND-1000 (NanoDrop technologies). DNA extracts of the three spatial replicates were pooled to get a composite sample per SU, as recommended in other reports [49].

Bacterial 16S rRNA genes were amplified with the primers W49 and W104-FAM labelled [50], [51]. For *Archaea*, we focused on the *Crenarchaeota* group because they have been reported as the most abundant and widely distributed group in terrestrial ecosystems [52]. Crenarcheal communities were assessed using primers targeting the 16S rRNA gene; 133FN6F-NED labelled and 248R5P [53]. Fungal ITS1 was amplified with the primers ITS5 and ITS2-HEX labelled [54].

PCR reactions (25 µl) contained 2.5 mM of MgCl_2_, 1X of AmpliTaq GoldTM buffer, 0.4 µg of bovine serum albumin, 0.1 mM of each dNTP, 0.26 mM of each primer, 2 U of AmpliTaqGold DNA polymerase (Applied Biosystems, Courtaboeuf, France) and 10 ng of DNA template. The PCR reaction was carried out as follows: an initial phase at 95°C (10 min), followed by 30 cycles at; 95°C (30 s), 56°C (15 s) and 72°C (15 s), and a final step at 72°C (7 min). PCR products were checked on a 1.5% agarose gel, and amplicons of each microbial community from the same SU were pooled to perform multiplex CE-SSCP.

PCR products were then submitted to CE-SSCP. Briefly, CE-SSCP consists in sorting DNA amplicons by electrophoresis under native conditions, according to their length and their nucleotide composition. Indeed, depending on their nucleotide composition, single-strand DNAs adopt secondary structures that vary in migration time under non-denaturing conditions. CE-SSCP is as robust as other fingerprinting methods but also more adapted to high-throughput analyses since (i) it avoids the use of harmful chemical for creating denaturing conditions and (ii) amplicons do not require any pre-treatment with restriction enzymes, decreasing considerably the experimental costs [55]. As other fingerprinting methods, SSCP produce fluorescence profiles where each peak represents the relative abundance of one or groups of microbial types. The overall SSCP profile is then used as a snapshot of the whole microbial community.

CE-SSCPs were performed on an ABI Prism 3130 XL genetic analyzer (Applied Biosystems, Courtaboeuf, France), as previously described in [55]. The resulting CE-SSCP profiles were normalized in order to reduce the variations of fluorescence level between profiles. We estimated microbial dissimilarity between any two SUs by calculating a Bray-Curtis index between Hellinger-transformed CE-SSCP profiles [56]. The resulting distance matrix is hereafter referred to as the microbial-dissimilarity matrix.

### Statistical analyses

Vegetation- and microbial-dissimilarity matrices were ordinated using Principal Coordinate Analysis (PCoA) [56]. We used a vector fitting method to identify directions in the floristic and microbial ordination space towards which a given environmental variable changes the most [57]. The vector fitting was tested for significance by means of 1000 permutations.

Distance-based redundancy analysis (db-RDAs) was used to test for the effect of different drivers on microbial assemblages. This approach provides the flexibility of choosing distance metrics more appropriate than Euclidean distances for community composition data [56]. We applied variance partitioning methods [44] to evaluate the relative contribution of the drivers on microbial assemblages. Explanatory variables included (i) plant species composition summarized by the first eight axes of the PCoA of the vegetation matrix that accounted for 74.9% of the variation, (ii) environmental variables (i.e. El, Arad, soil pH, SOM and LP), and (iii) geographical distance. Significance was tested using partial db-RDAs with 1000 Monte Carlo permutations. All statistical analyses were carried out with the R software [58]. Vector fitting, db-RDAs and variation partitioning were conducted with the R package vegan [57].

## Supporting Information

Figure S1 **Variation partitioning based on db-RDAs models with different plant community descriptors.** (**a**) Variation explained by each vegetation-PCoA eigenvectors. Variation of crenarchaeal (**b**), bacterial (**c**) and fungal (**d**) communities explained by pure and combined effects of P: plant communities as defined by different numbers of vegetation PCoA eigenvectors (x axis), E: environmental conditions (i.e. El, Arad, pH, SOM and LP, See Fig. 2 for abbreviations) and S: geographic distances. The significance of the full model (All) and the pure effects was assessed by using 1000 Monte Carlo permutations, and is indicated with solid symbols. NA: not applicable. The vertical black arrows indicates the model used in Fig. 4, Table S2.
(TIF)

Figure S2 **Spatial correlogram of crenarchaeal (a), bacterial (b) and fungal (c) community dissimilarities based on Mantel test and Spearman's ρ coefficient.** Distance classes are indicated in meters. Open squares indicate that all estimates are non-significant (1000 Monte Carlo permutations, Bonferroni-corrected *P*>0.05).
(TIF)

Table S1 **Environmental characteristics of sampling units (SUs).**
(DOC)

Table S2 **Relative contribution of plant community composition, environment and geographic distances in the variation of crenarchaeal, bacterial and fungal communities.**
(DOC)
