# Supplementary figures and images for: Contrasting Diversity Patterns of Crenarchaeal, Bacterial and Fungal Soil Communities in an Alpine Landscape
Source: PLoS One. 2011 May 12;6(5):e19950. doi: 10.1371/journal.pone.0019950 (PMC3093402; doi:10.1371/journal.pone.0019950)

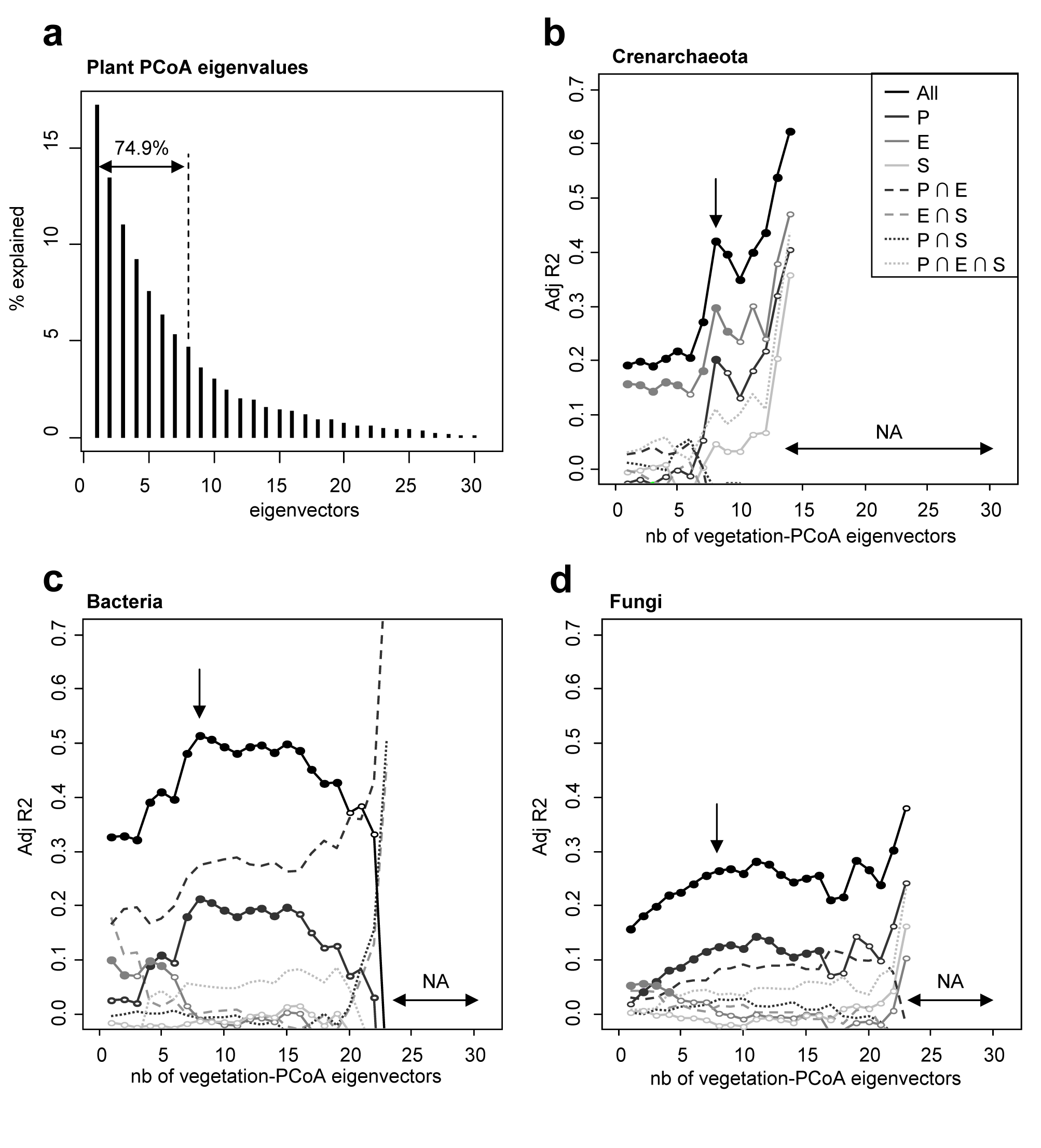

Supplement: Figure S1 — Variation partitioning based on db-RDAs models with different plant community descriptors. (a) Variation explained by each vegetation-PCoA eigenvectors. Variation of crenarchaeal (b), bacterial (c) and fungal (d) communities explained by pure and combined effects of P: plant communities as defined by different numbers of vegetation PCoA eigenvectors (x axis), E: environmental conditions (i.e. El, Arad, pH, SOM and LP, See Fig. 2 for abbreviations) and S: geographic distances. The significance of the full model (All) and the pure effects was assessed by using 1000 Monte Carlo permutations, and is indicated with solid symbols. NA: not applicable. The vertical black arrows indicates the model used in Fig. 4, Table S2. (TIF) [file pone.0019950.s001.tif]

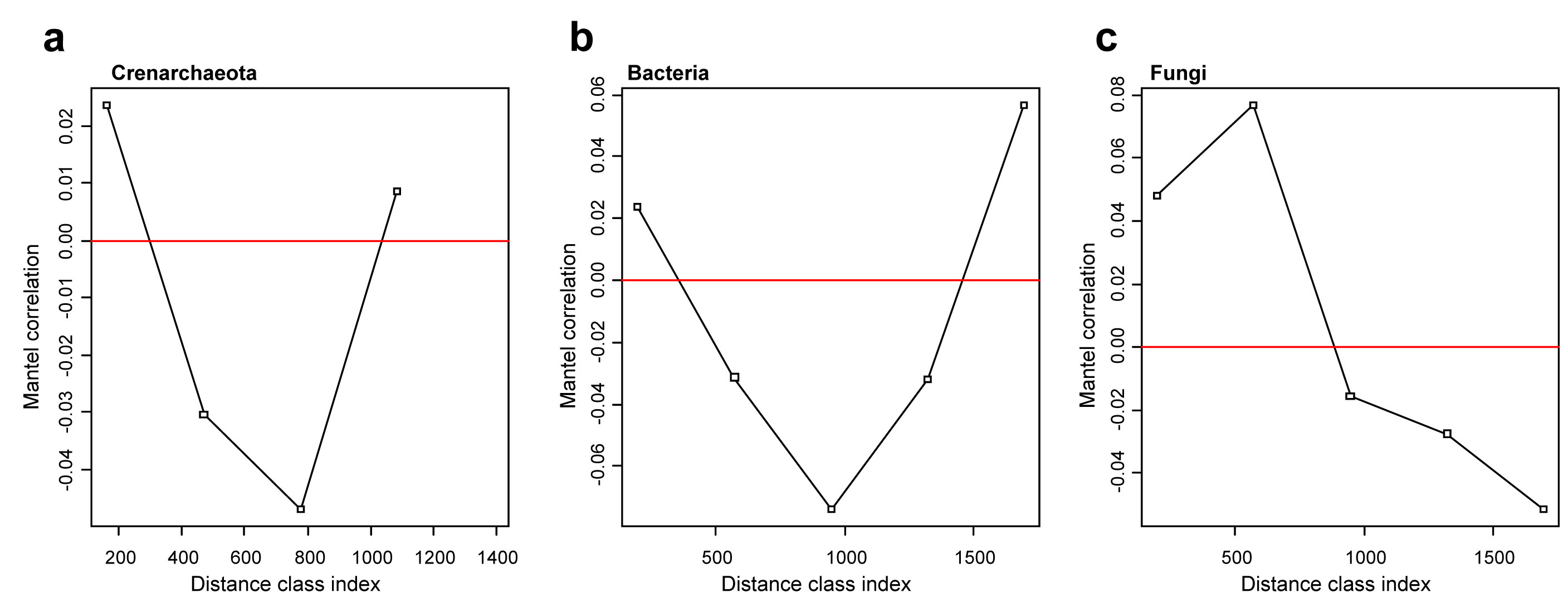

Supplement: Figure S2 — Spatial correlogram of crenarchaeal (a), bacterial (b) and fungal (c) community dissimilarities based on Mantel test and Spearman's ρ coefficient. Distance classes are indicated in meters. Open squares indicate that all estimates are non-significant (1000 Monte Carlo permutations, Bonferroni-corrected P>0.05). (TIF) [file pone.0019950.s002.tif]
