# Supplementary material for: Contrasting Diversity Patterns of Crenarchaeal, Bacterial and Fungal Soil Communities in an Alpine Landscape
Source: PLoS One. 2011 May 12;6(5):e19950. doi: 10.1371/journal.pone.0019950 (PMC3093402; doi:10.1371/journal.pone.0019950)
Supplement: Table S1 — Environmental characteristics of sampling units (SUs). (DOC) [file pone.0019950.s003.doc]

**Table S1: Environmental characteristics of sampling units (SUs).**

| SUs | Latitude (dd)^#^ | Longitude (dd)^#^ | Elevation (m a.s.l.)^§^ | Slope (°) | Orientation (°) | Annual Radiations (kJ m^-2^d^-1^) | pH | SOM (%) | Aboveground Phytomass  (g m^-^²)^$^ |
| --- | --- | --- | --- | --- | --- | --- | --- | --- | --- |
| CF31 | 45.050 | 6.385 | 2502 | 8.5 | -1.0 | 2022 | 5.0 | 8.9 | 195.0 |
| CF32 | 45.054 | 6.375 | 2571 | 9.0 | 0.2 | 3258 | 5.5 | 13.9 | 210.4 |
| CF33 | 45.054 | 6.378 | 2529 | 12.0 | 0.7 | 2655 | 5.1 | 10.4 | 278.8 |
| CTR11 | 45.051 | 6.383 | 2527 | 11.7 | -1.0 | 2200 | 4.8 | 34.8 | 250.8 |
| CTR12 | 45.057 | 6.378 | 2553 | 12.0 | -0.6 | 8067 | 5.1 | 25.7 | 246.2 |
| CTR13 | 45.054 | 6.392 | 2436 | 19.3 | -1.0 | 9326 | 5.0 | 26.6 | 180.2 |
| ES57 | 45.066 | 6.366 | 2818 | 19.5 | -0.7 | 8933 | 8.0 | 2.8 | 161.8 |
| ES58 | 45.058 | 6.391 | 2586 | 31.1 | -0.7 | 10050 | 7.5 | 2.7 | 38.9 |
| FG61 | 45.056 | 6.372 | 2672 | 24.4 | 0.1 | 7157 | 6.1 | 27.0 | 414.8 |
| FG62 | 45.062 | 6.365 | 2812 | 37.1 | 0.1 | 8749 | 6.1 | 14.9 | 511.6 |
| FG63 | 45.064 | 6.373 | 2723 | 41.5 | -1.0 | 10991 | 5.9 | 8.8 | 243.0 |
| FP1 | 45.057 | 6.385 | 2486 | 26.6 | -0.8 | 11186 | 5.4 | 24.3 | 2300.9 |
| FP2 | 45.057 | 6.389 | 2440 | 27.2 | -0.9 | 9695 | 5.5 | 19.7 | 2470.9 |
| FP3 | 45.057 | 6.382 | 2445 | 31.3 | -1.0 | 9101 | 5.6 | 18.2 | 2107.6 |
| HS21 | 45.056 | 6.392 | 2508 | 35.3 | -0.7 | 10605 | 7.0 | 6.7 | 146.6 |
| HS22 | 45.059 | 6.387 | 2586 | 28.3 | -0.9 | 12531 | 6.4 | 15.5 | 145.5 |
| HS23 | 45.054 | 6.395 | 2449 | 33.0 | -1.0 | 11604 | 7.3 | 7.4 | 138.2 |
| KD41 | 45.050 | 6.385 | 2503 | 14.0 | -1.0 | 2096 | 6.4 | 17.4 | 314.3 |
| KD42 | 45.054 | 6.378 | 2528 | 8.1 | 0.6 | 2642 | 6.1 | 25.2 | 228.1 |
| KD43 | 45.055 | 6.375 | 2579 | 14.0 | 0.5 | 4218 | 6.1 | 23.8 | 298.7 |
| KS46 | 45.056 | 6.372 | 2646 | 26.6 | 0.0 | 6343 | 7.2 | 18.5 | 430.3 |
| KS47 | 45.060 | 6.365 | 2749 | 12.6 | -0.5 | 10969 | 5.9 | 26.0 | 359.6 |
| KS48 | 45.059 | 6.368 | 2693 | 18.5 | 0.5 | 7779 | 6.2 | 29.7 | 408.4 |
| SR36 | 45.048 | 6.389 | 2499 | 28.3 | 0.9 | 1386 | 7.5 | 18.9 | 307.2 |
| SR37 | 45.053 | 6.380 | 2534 | 29.2 | 0.9 | 2258 | 7.5 | 20.3 | 191.2 |
| SR38 | 45.053 | 6.375 | 2595 | 31.1 | 0.9 | 3590 | 6.7 | 18.9 | 233.2 |
| TR6 | 45.059 | 6.389 | 2615 | 31.3 | -1.0 | 11374 | 6.2 | 9.2 | 497.8 |
| TR7 | 45.053 | 6.394 | 2380 | 19.3 | -1.0 | 8451 | 6.4 | 21.1 | 403.7 |
| TR8 | 45.054 | 6.395 | 2417 | 31.1 | -1.0 | 10561 | 6.3 | 7.9 | 758.0 |
| V26 | 45.044 | 6.402 | 2227 | 17.8 | 0.7 | 6171 | 5.1 | 30.6 | 650.2 |
| V27 | 45.048 | 6.390 | 2467 | 36.4 | 0.5 | 1487 | 5.4 | 27.6 | 219.8 |
| V28 | 45.050 | 6.392 | 2333 | 24.2 | 1.0 | 2276 | 5.9 | 29.3 | 298.8 |

^#^ Decimal degree, ^§^ Meters above sea level, ^$^ Phytomass are dried mass.
