# Supplementary material for: Contrasting Diversity Patterns of Crenarchaeal, Bacterial and Fungal Soil Communities in an Alpine Landscape
Source: PLoS One. 2011 May 12;6(5):e19950. doi: 10.1371/journal.pone.0019950 (PMC3093402; doi:10.1371/journal.pone.0019950)
Supplement: Table S2 — Relative contribution of plant community composition, environment and geographic distances in the variation of crenarchaeal, bacterial and fungal communities. (DOC) [file pone.0019950.s004.doc]

**Table S2: Relative contribution of plant community composition, environment and geographic distances in the variation of crenarchaeal, bacterial and fungal communities.**

| Effects | Crenarchaeota | | Bacteria | | Fungi | |
| --- | --- | --- | --- | --- | --- | --- |
|  | Adj- R2 | *P*-value | Adj- R2 | *P*-value | Adj- R2 | *P*-value |
| P | 0.20 | 0.018 | 0.21 | <2.22e-16 | 0.12 | 0.001 |
| E | 0.30 | 0.010 | -0.01 | 0.583 | 0.00 | 0.472 |
| S | 0.05 | 0.190 | -0.01 | 0.726 | -0.02 | 0.750 |
| P∩E | -0.11 |  | 0.28 |  | 0.08 |  |
| E∩S | -0.08 |  | 0.00 |  | 0.01 |  |
| P∩S | -0.04 |  | -0.01 |  | 0.03 |  |
| P∩E∩S | 0.11 |  | 0.05 |  | 0.04 |  |
| Total Model | 0.42 | <2.22e-16 | 0.51 | <2.22e-16 | 0.26 | <2.22e-16 |
| Residuals | 0.58 |  | 0.49 |  | 0.74 |  |

Variation in the community table was partitioned according to P: eight first PCoA axes obtained from plant distance matrix based on Bray-Curtis distances and Hellinger-transformed data; E: environmental parameter as used before; S: space as Euclidean geographic coordinates. The values indicated here are represented in Fig. 4 and correspond to Adjusted R^2^. Significance was tested using partial db-RDAs with 1000 Monte Carlo permutations. Combined effects are not testable for significance. NA: not applicable.
